# Multimodal characterisation of spontaneous Merkel cell carcinoma in the endangered Caucasian squirrel *(Sciurus anomalus pallescens):* integrating spatial transcriptomics, imaging mass cytometry and metagenomic sequencing

**DOI:** 10.2478/jvetres-2026-0034

**Published:** 2026-06-30

**Authors:** Peyman Mohammadzadeh, Amin Pilvaieh, Arshia Dousti, Mohammad Reza Salim Bahrami, Farshad Ziaee

**Affiliations:** 1 Department of Pathology, Sa. C. Islamic Azad University, Sanandaj, Iran; 2 Student Research Committee, Tabriz University of Medical Sciences, Tabriz, Iran

**Keywords:** comparative oncology, imaging mass cytometry, Merkel cell carcinoma, spatial transcriptomics, wildlife disease

## Abstract

**Introduction:**

Merkel cell carcinoma is an aggressive neuroendocrine skin malignancy rarely reported in non-domestic species.

**Material and Methods:**

A cutaneous nodule from an endangered Caucasian squirrel (*Sciurus anomalus pallescens*) was examined using histopathology, immunohistochemistry, imaging mass cytometry, spatial transcriptomics (10× Visium) and metagenomic sequencing.

**Results:**

Histology revealed a high-grade neuroendocrine carcinoma with frequent mitoses (52 per 2.37 mm^2^) and necrosis. Tumour cells were positive for cytokeratin 20 (paranuclear dot pattern), synaptophysin and chromogranin A, with a high Ki-67 index (68%). Spatial analyses delineated a distinct tumour core and combined invasive front and stromal compartments, revealing upregulation of neuroendocrine (atonal basic helix–loop–helix transcription factor 1 and neurogenic differentiation factor 1) and proliferative (marker of proliferation Ki-67) programmes, and activation of phosphoinositide 3-kinase–AKT serine/threonine kinase 1–mechanistic target of rapamycin and mitogen-activated protein kinase pathways. No evidence of Merkel cell polyomavirus was found. The tumour microenvironment was immune-excluded, with programmed-death ligand 1 expression on ~22% of tumour cells and CD8^+^ T cells restricted to the stroma.

**Conclusion:**

This study provides a comprehensive methodological framework for high-resolution tumour profiling in conservation pathology and highlights the emergence of neoplasia in threatened wildlife.

## Introduction

Merkel cell carcinoma (MCC) is a highly aggressive cutaneous neuroendocrine malignancy characterised by rapid growth, early metastasis and poor clinical outcomes in humans (6, 36). Two major biological subtypes are recognised: Merkel cell polyomavirus (MCPyV)-positive tumours, which demonstrate viral oncoprotein expression and relatively low mutational burden, and MCPyV-negative tumours, which exhibit extensive UV-associated DNA damage, genomic instability and activation of proliferative signalling pathways (35). Beyond humans, MCC has been reported only sporadically in domestic animals, including dogs and cats, but its true incidence, aetiologies and molecular signatures across non-domestic species remain poorly understood (14, 16, 39). The near absence of documented cases in wildlife represents a major gap in comparative oncology, especially given the increasing recognition that neoplastic diseases are emerging health threats in threatened and endangered species (26). The Caucasian squirrel (*Sciurus anomalus pallescens*) is a regionally endangered sciurid with declining populations in parts of its native range. Despite its conservation importance, little is known about its susceptibility to neoplastic or inflammatory diseases. Documentation of spontaneous malignancies in such species holds dual significance: first, it expands the phylogenetic and biological spectrum of tumour types; and second, it provides critical insight into potential environmental, infectious or genetic drivers relevant to conservation medicine (29). The discovery of MCC in this species therefore presents a unique opportunity to investigate neuroendocrine tumourigenesis in a wild rodent host using modern molecular and spatial tools not previously applied in wildlife oncology. Recent technological advances – including spatial transcriptomics, imaging mass cytometry and high-resolution metagenomic sequencing – now enable detailed, *in-situ* characterisation of tumour ecosystems at unprecedented resolution (9, 21). Spatial transcriptomics preserves tissue architecture while mapping gene expression within discrete anatomical niches, allowing delineation of lineage programmes, proliferative zones and immune exclusion patterns. Imaging mass cytometry complements this by resolving multi-marker protein expression at single-cell resolution across the tumour stroma interface, defining the phenotypic and spatial organisation of immune infiltrates and stromal elements. Concurrent metagenomic sequencing and targeted molecular assays facilitate sensitive detection or exclusion of viral contributors, particularly relevant in MCC where MCPyV status holds major biological and mechanistic implications (43). To date, no comprehensive multimodal characterisation of MCC has been reported in any wild mammalian species. The absence of such data limits understanding of tumour biology across evolutionary contexts and impedes recognition of oncologic threats in vulnerable wildlife populations. Here, the first molecularly and spatially resolved case of spontaneous MCC in the endangered Caucasian squirrel is presented. By integrating histopathology, immunophenotyping, spatial transcriptomics, imaging mass cytometry and metagenomic viral screening, this study establishes a comprehensive diagnostic profile of MCC in this species, determines its MCPyV status and associated molecular pathways and characterises the spatial architecture of the tumour microenvironment. This work expands the comparative oncology repertoire of MCC and provides a methodological framework for high-resolution tumour profiling in conservation pathology

## Material and Methods

### Case description and sampling

A spontaneous cutaneous mass was identified in an adult male Caucasian squirrel (*Sciurus anomalus pallescens*) that died naturally in a zoological conservation facility in western Iran. The region is characterised by a temperate mountainous climate with average annual precipitation of 450 mm and seasonal temperature variations ranging from –5°C in winter to 35°C in summer. The vegetation primarily consists of oak forests interspersed with almond and pistachio woodlands. The facility housed the individual in a naturalistic enclosure designed to mimic native habitat conditions until its natural death.

A complete diagnostic necropsy was performed within 4 h of death. A solitary cutaneous nodule measuring 5.2 cm in diameter was excised. Representative tissues from the tumour and major organs were fixed in 10% neutral-buffered formalin for 24–36 h and processed routinely. Additional tumour fragments were snap frozen in liquid nitrogen for downstream molecular analyses. Given the large size of the lesion, sufficient tumour material was available to support all multimodal downstream assays including spatial transcriptomics, imaging mass cytometry, proteomics and viral metagenomics without compromising the integrity of any platform. All procedures complied with approved wildlife ethics guidelines of the supervising institution (Eram Zoo Wildlife Ethics Committee Protocol No. EZ WE 2025 019).

### Histopathology, mitotic count, and assessment of necrosis and lymphovascular invasion

Formalin-fixed tissues were embedded in paraffin, sectioned at 4 μm and stained with HE. Periodic acid–Schiff and Giemsa stains (Sigma Aldrich, Gillingham, UK) were performed according to standard protocols to evaluate basement membrane integrity, mast cell density and microbial colonisation. Whole slide images were acquired using a Hamamatsu NanoZoomer S360 scanner at 40× magnification with 0.23 μm/pixel resolution (Hamamatsu Photonics, Hamamatsu, Japan). Mitotic figures were enumerated using a microscope in non-overlapping 400× high-power fields within the most mitotically active hotspot, covering a total area of 2.37 mm^2^ (equivalent to 10 contiguous 400× fields with a field number (FN) 22 mm ocular), consistent with recommended international standards for mitotic count methodology in veterinary oncology (23, 41). Areas with necrosis, haemorrhage or oedema were systematically excluded. Tumour necrosis was quantified histologically using optical estimation guidelines outlined by the Veterinary Cancer Guidelines and Protocols group (17). Lymphovascular invasion was assessed using strict criteria, requiring unequivocal tumour cell emboli within endothelium-lined spaces (30).

### Morphometry

Quantitative morphometric analysis was performed using the HALO image analysis platform v. 4.3 (Indica Labs, Albuquerque, NM, USA) and QuPath v. 0.5.1 (2), utilising artificial intelligence-based nuclear segmentation and tissue classification algorithms. Measured parameters included nuclear cross-sectional area (μm^2^), chromatin texture features (Haralick features), mitotic figure density (per mm^2^), Ki-67 labelling index (%), and nuclear-to-cytoplasmic ratio.

### Transmission electron microscopy

Tumour tissue samples fixed in 2.5% glutaraldehyde in 0.1 M phosphate buffer were post-fixed in 1% osmium tetroxide, dehydrated through a graded ethanol series and embedded in EPON 812 resin (Ted Pella, Reading, CA, USA). Ultrathin sections (70 nm) were cut using a diamond knife (DiATOME, Quakertown, PA, USA) on an EM UC7 ultramicrotome (Leica Biosystems, Nussloch, Germany), stained with uranyl acetate and lead citrate and examined using a Talos F200X transmission electron microscope operating at 200 kV (Thermo Fisher Scientific, Eindhoven, the Netherlands). Ultrastructural analysis focused on identifying densecore neurosecretory granules (50–150 nm diameter), paranuclear intermediate filament aggregates and mitochondrial morphology.

### Multiplex immunofluorescence validation

Multiplex immunofluorescence was performed using the OPAL 7 Color Automation IHC (immunohistochemistry) Kit (Akoya/Quanterix, Billerica, MA, USA) on the Leica BOND RX automated staining platform. The antibody panel included: cytokeratin 20 (clone Ks20.8; Dako/Agilent, Santa Clara, CA, USA, used at 1 : 100), synaptophysin (clone SP11; Thermo Fisher Scientific, Waltham, MA, USA, used at 1 :200), chromogranin A (clone LK2H10, Thermo Fisher Scientific, used at 1 :300), insulinoma-associated protein 1 (INSM1) (clone A-8; Santa Cruz Biotechnology, Dallas, TX, USA, used at 1 :250), Ki-67 (clone MIB-1, Dako/Agilent, used at 1 :200) and programmed-death ligand 1 (PD-L1) (clone 22C3; Agilent, used at 1 : 150). Sequential antibody application, microwave-based epitope retrieval and tyramide signal amplification were performed according to the manufacturer’s protocols. Multispectral imaging was acquired using a Vectra Polaris automated quantitative pathology imaging system (Akoya/Quanterix). Protein co-localisation analysis was performed using inForm v. 2.8 image analysis software (Akoya/Quanterix).

### Antibody cross-reactivity validation

Comprehensive antibody validation was performed following international guidelines for cross-species immunohistochemistry (28). Validation procedures included: (i) Western blot analysis of squirrel tumour and normal tissue lysates extracted in radio-immunoprecipitation assay buffer with cOmplete Protease Inhibitor Cocktail (Roche, Basel, Switzerland), (ii) protein quantification *via* bicinchoninic acid assay (Pierce/Thermo Fisher Scientific, Rockford, IL, USA), (iii) electrophoretic separation on 4–12% Bis-Tris NuPAGE gels (Invitrogen, Carlsbad, CA, USA) and (iv) peptide blocking assays for cytokeratin 20 (CK20) and synaptophysin to confirm antigen specificity. Additional immunohistochemical validation was performed on custom-constructed human–squirrel tissue microarrays containing 24 cores (12 human and 12 squirrel).

### Liquid chromatography–tandem mass spectrometry proteomic analysis

Frozen tumour tissue and matched normal skin samples (n = 3 biological replicates each) were homogenised in 8 M urea lysis buffer (100 mM Tris-HCl, pH 8.5) containing Halt Protease and Phosphatase Inhibitor Cocktail (Pierce/Thermo Fisher Scientific). Proteins were reduced with 5 mM dithiothreitol for 30 min at 37°C, alkylated with 15 mM iodoacetamide for 20 min at room temperature in darkness and digested overnight with sequencing-grade modified trypsin (Promega, Madison, WI, USA) in 1 :50 proportion (w/w) at 37°C. Peptides were desalted using Sep-Pak C18 cartridges (Waters, Milford, MA, USA) and analysed on an Orbitrap Eclipse Tribrid mass spectrometer (Thermo Fisher Scientific, Eindhoven, the Netherlands) coupled to an UltiMate 3000 RSLCnano system (Dionex/Thermo Fisher Scientific, Sunnyvale, CA, USA). Data-dependent acquisition was performed with 120,000 resolution for MS1 and 30,000 resolution for MS2 scans. Raw spectra were processed using MaxQuant v. 2.2.1.0 (5) against a custom *S. anomalus* protein database with 18,542 entries. Label-free quantification and statistical analysis were performed in Perseus v. 2.0.9.0 (38), with pathway enrichment analysis *via* ReactomePA v. 1.52.0 (44) and Ingenuity Pathway Analysis (QIAGEN, Hilden, Germany).

### Viral metagenomics and qPCR

Metagenomic assemblies were generated using MEGAHIT v. 1.2.9 (20) for k-mer sizes 21–141 and annotated using DIAMOND v. 2.1.9 (4) against release 229 of the NCBI Viral RefSeq database. Phylogenetic analysis was performed with IQ-TREE v. 2.3.5 (24) using the ModelFinder option and 1,000 ultrafast bootstrap replicates. Recombination analysis was conducted using RDP5 (recombination detection program) v. 5.5 (22). Merkel cell polyomavirus-specific TaqMan qPCR assays targeting conserved large T-antigen regions and using a 5′-CAGGTCCCCACTACTATTGC-3′ forward primer, 5′-GGTGCTGCTTCAGTAGTAGC-3′ reverse primer and FAM-CCACCAACCTCT-MGB probe were performed on a QuantStudio 7 Flex Real-Time PCR System (Applied Biosystems, Foster City, CA, USA), with absolute quantification using a standard curve of cloned target sequences (10^2^–10^8^ copies/reaction).

### Integrative bioinformatics analysis

Multiomics data integration was performed using R v. 4.4.0 (27) with Seurat v. 5.1.0 (14) for transcriptomic analysis, limma (linear models for microarray data) v. 3.62.2 (31) for differential expression, ReactomePA v. 1.52.0 for pathway enrichment and ComplexHeatmap v. 2.24.0 (13) for data visualisation. Ligand–receptor interactions were analysed using CellPhoneDB v.5.0.0 (37) and spatial colocalisation was quantified using Ripley’s K-function over a radius range of 0–100 μm. Network analysis and visualisation were performed in Cytoscape v. 3.10.3 (34) with the cytoHubba plugin (7).

### Immunohistochemistry and antibody validation

Immunohistochemistry was performed on a Leica BOND RX system using primary antibodies against CK20, synaptophysin, chromogranin A, INSM1, Ki-67 and PD-L1. Heat-induced epitope retrieval was performed using ER2 (epitope retrieval solution 2) buffer (pH 9.0). Detection utilised a polymer-based HRP system with 3,3′-diaminobenzidine chromogen. Positive controls included validated human Merkel cell carcinoma tissue and species-matched normal squirrel tissues when available.

Because most antibodies were raised against human antigens, cross-species validation followed the recommendations of Ramos-Vara *et al*. (28). Western blot analysis confirmed antibody specificity and cross-reactivity with squirrel antigens, showing bands at expected molecular weights in tumour lysates (Fig. 1). Proteins (30 μg per lane) were separated by SDS-PAGE under reducing conditions on 4–12% Bis-Tris gels and transferred to polyvinylidene fluoride membranes. Blots were probed with primary antibodies (CK20 (clone Ks20.8 used at 1 :1,000), synaptophysin (clone SP11 used at 1 :2,000) and chromogranin A (clone LK2H10 used at 1 : 1,500)), next with HRP-conjugated secondary antibodies used at 1:5,000, and finally chemiluminescent detection was undertaken with SuperSignal West Pico PLUS substrate (Pierce/Thermo Fisher Scientific). The proliferation index of Ki-67 was calculated using automated nuclear detection (HALO; Indica Labs) across at least 1,000 tumour cells in the most proliferative regions (16).

**Fig. 1.**
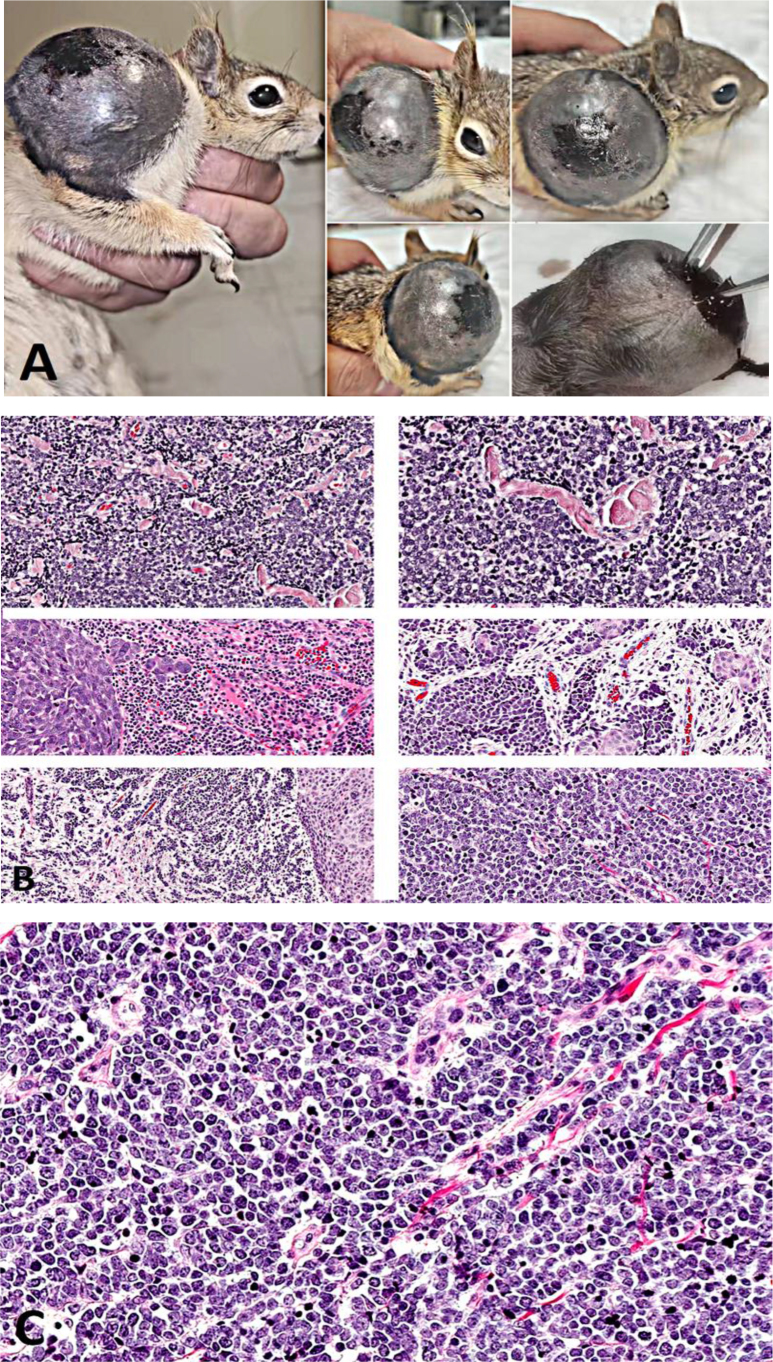

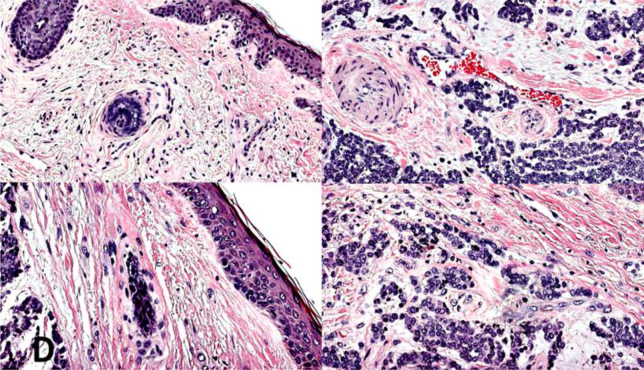
Gross and histopathological features of spontaneous Merkel cell carcinoma in *Sciurus anomalus pallescens*. (A) Gross appearance of the excised lesion showing a firm, dome-shaped dermal nodule with pale tan surface and multifocal haemorrhagic foci. (B) Histopathological examination of the tumour showing solid sheets and trabeculae of monomorphic small round-to-oval cells with scant cytoplasm and hyperchromatic nuclei (HE). (C) Mitotic count of the tumour showing mitotic figures indicative of high proliferative activity (HE). (D) Lymphovascular invasion in multiple foci, with tumour emboli enclosed by an intact endothelial lining, fulfilling Veterinary Cancer Guidelines and Protocols diagnostic criteria (HE)

### Spatial transcriptomics

Spatial gene expression profiling was performed using the 10× Genomics Visium platform (10× Genomics, Pleasanton, CA, USA). Optimal cutting temperature compound-embedded tumour tissue was cryosectioned at 10 μm, HE stained and imaged. Ribonucleic acid integrity was confirmed prior to library preparation. Spatially barcoded cDNA libraries were generated according to manufacturer protocols and sequenced on an Illumina NovaSeq 6000 system (Illumina, San Diego, CA, USA). Raw FASTQ files were processed using Space Ranger v. 2.1.1 (10× Genomics). Downstream analyses – including clustering, differential expression and pathway enrichment – were performed in Seurat v. 4.3 (14).

### Imaging mass cytometry

This was performed on a Fluidigm Hyperion Imaging System (Standard BioTools, San Francisco, CA, USA). Formalin-fixed, paraffin-embedded tissue sections 4 μm thick were stained with a metal-tagged antibody panel targeting tumour (CK20, synaptophysin and INSM1), immune (CD3, CD8, CD68 and PD-L1), and stromal (alpha smooth-muscle actin and vimentin) markers. Data were acquired by laser ablation, and single-cell segmentation and spatial analysis were performed using MCD Viewer (Standard BioTools) and histoCAT software (18, 32). Immunoscore IC analysis was performed using the Immunoscore IC software (HalioDx, now Veracyte, Marseille, France) for immune checkpoint scoring.

### Viral metagenomics and MCPyV screening

Total DNA was extracted using a phenol-chloroform protocol (8, 19). Viral metagenomic profiling was conducted using MEGAHIT v. 1.2.9 for assembly and DIAMOND v. 2.1.9 against viral RefSeq for annotation. Presence of Merkel cell polyomavirus was assessed using a TaqMan qPCR assay targeting conserved T-antigen sequences, following validated MCPyV detection methods. All procedures were conducted in accordance with approved institutional wildlife ethics protocols and complied with national and institutional guidelines for the care and use of animals.

### Cell line authentication statement

No established or primary cell lines were used in this study. All analyses were performed exclusively on primary tumour tissue obtained from a naturally occurring MCC in *Sciurus anomalus pallescens*. Therefore, cell line authentication, mycoplasma testing and short tandem repeat profiling are not applicable to this work.

## Results

### Gross and histopathological findings

A well-circumscribed but unencapsulated dermal-subcutaneous mass measuring 5.2 × 0.9 cm was identified on the lateral thoracic region (Fig. 1A). The longer axis (5.2 cm) corresponded to the clinically described diameter of the lesion, while the second dimension represented its maximal thickness, resolving the apparent discrepancy in tumour measurements. The cut surface was solid, pale tan and focally haemorrhagic. Histologically, the tumour consisted of densely packed sheets, trabeculae and nests of small round-to-polygonal cells with scant eosinophilic cytoplasm, finely granular (“salt and pepper”) chromatin and inconspicuous nucleoli (Fig. 1B). Mitotic figures were numerous, including atypical forms, with a mitotic count of 52 per 2.37 mm^2^ (Fig. 1C). Geographic necrosis was prominent, occupying approximately 18% of the total tumour area (Table 1). Lymphovascular invasion was identified at multiple foci, characterised by intraluminal tumour emboli in endothelium-lined spaces (Fig. 1D).

**Table 1.** Quantitative morphometric analysis of the Merkel cell carcinoma in *Sciurus anomalus pallescens*

Parameter	Methodology / quantification approach	Measured value (mean ± SD)	Interpretation / diagnostic significance
Total tumour area (mm^2^)	Digital planimetry; threshold-based segmentation of tumour parenchyma vs. stroma	12.86 ± 0.41	Defines baseline morpho-metric field for necrosis and mitotic density assessment
Necrotic area (% of tumour parenchyma)	Morphometric segmentation of necrotic zones (eosinophilic debris, nuclear karyorrhexis and ghost cell outlines) relative to viable tumour tissue	18.2 ± 1.3%	Consistent with extensive ischaemic necrosis typical of high grade neuroendocrine carcinomas
Mitotic count (per 2.37 mm^2^)	Enumerated manually under 400× magnification (field number 22 mm ocular) across 10 contiguous high-power fields	52	Reflects markedly elevated proliferative index, supporting Grade III malignancy
Ki-67 labelling index (% of tumour nuclei)	Immunohistochemical quantification using QuPath v. 0.4.3 (n = 2,500 cells and threshold ≥ 1% nuclear staining) following established digital image analysis guidelines for Ki-67 assessment in neuroendocrine tumours (3)	68.1 ± 2.4%	Indicates extreme proliferative activity and high cellular turnover rate
Lymphovascular invasion	Identified morphologically and verified *via* CD31 immunostaining; strict Veterinary Cancer Guidelines and Principles criteria applied	Present (3 foci / section)	Confirms intraluminal tumour emboli with intact endothelial lining; predictor of metastasis
Tumour-stroma ratio	Ratio of tumour cellularity to stromal area (imaging mass cytometry segmentation, single-cell annotation and 1,000-μm^2^ regions of interest)	3.4:1	Demonstrates high tumour cell density and limited stromal buffering capacity
Mean nuclear diameter (μm)	Quantified on HE images (n = 200 cells) using HALO CytoNuclear module	8.3 ± 0.6	Reflects monomorphic small-cell morphology characteristic of MCC
Tumour necrosis index	Composite score = (necrotic area × mitotic count) / total viable tumour area	0.73 ± 0.04	Quantitative index correlating with high-grade neuro-endocrine tumour aggressiveness

Morphometric and immunohistochemical analyses were performed according to Veterinary Cancer Guidelines and Protocols (23). Quantification of Ki-67 followed digital pathology guidelines for neuroendocrine tumours (3) and adhered to standardised international units for mitotic area (2.37 mm^2^ = 10 contiguous high-power fields, 400× magnification and field number 22 mm). All morphometric metrics represent mean ± SD derived from three independent tumour sections.

### Western blot

This analysis demonstrated specific bands at the expected molecular weights for CK20, synaptophysin and chromogranin A in tumour lysates, consistent with the human positive control, with no signal in negative controls, confirming antibody specificity and cross-reactivity (Fig. 2). Full-length uncropped blots are provided in Supplementary Materials.

**Fig. 2.**
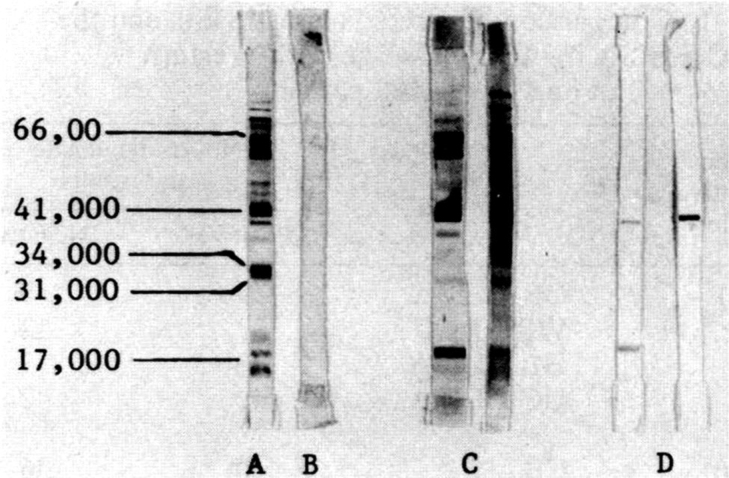
Western blot validation of antibody cross-reactivity with Sciurus anomalus antigens. (A) Molecular weight marker (kDa) and human Merkel cell carcinoma (MCC) positive control tissue lysate showing expected bands for cytokeratin 20 (~46 kDa), synaptophysin (~38 kDa), and chromogranin A (~48–55 kDa). (B) Negative control (no primary antibody) confirming absence of non-specific signal. (C) *Sciurus anomalus pallescens* normal skin lysate demonstrating weak or absent target protein expression, consistent with tissue specificity. (D) *Sciurus anomalus pallescens* MCC tumour lysate displaying specific bands corresponding to the expected molecular weights observed in the human positive control, confirming antibody cross-reactivity

### Immunophenotypic characterisation

Immunohistochemistry demonstrated strong cytoplasmic expression of synaptophysin and chromogranin A, along with strong nuclear positivity for INSM1 (Fig. 3A). Cytokeratin 20 exhibited the characteristic paranuclear dot-like distribution (Fig. 3B). The Ki-67 proliferation index was markedly elevated at 68% (Fig. 3C). Programmed-death ligand 1 expression was observed in ~22% of tumour cells (Fig. 4). Only scattered perivascular CD3-positive T lymphocytes and rare CD20-positive B cells were detected.

**Fig. 3.**
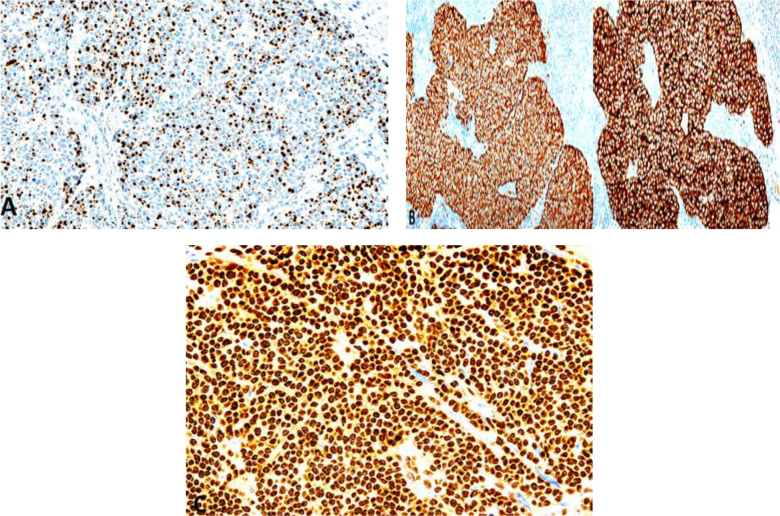
Immunohistochemical characterisation of the Merkel cell carcinoma in *Sciurus anomalus pallescens*. (A) Cytokeratin 20 immunohistochemistry showing characteristic paranuclear dot-like pattern. (B) Robust expression of chromogranin A and synaptophysin, confirming neuroendocrine differentiation. (C) Ki-67 labelling index of 68%, indicating high proliferative activity

**Fig. 4.**
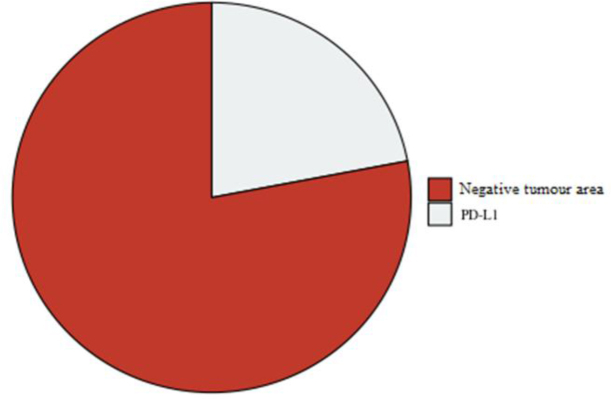
Programmed-death ligand 1 (PD-L1) expression detection in neoplastic cells in *Sciurus anomalus pallescens*, indicative of immune evasion mechanisms

### Spatial transcriptomic profiling

Spatial transcriptomics generated 1,726 high-quality spots across the tumour section (Fig. 5A). Only spots containing ≥500 unique molecular identifiers and ≥300 detected genes were retained, resulting in a final dataset of 1,726 high-confidence spots after stringent quality filtering; lower quality peripheral spots were excluded to prevent artefactual clustering. Unsupervised clustering delineated a central proliferative neuroendocrine domain characterised by high expression of INSM1, atonal basic helix–loop–helix transcription factor (ATOH1), marker of proliferation Ki-67, DNA topoisomerase II alpha and achaete-scute family basic helix–loop–helix transcription factor 1 (Fig. 5B). A stromal cluster exhibited enriched expression of collagen type I alpha 1 chain, collagen type III alpha 1 chain and platelet-derived growth factor receptor alpha, consistent with fibroinflammatory matrix remodelling (Fig. 5D). The peripheral immune-associated zone contained low-level CD3 delta chain and CD8 alpha chain transcripts but lacked robust activation signatures. Ligand-receptor analysis demonstrated reduced C-X-C motif chemokine ligand 9/ligand 10 (CXCL9/10)–C-X-C motif chemokine receptor 3 (CXCR3) signalling and stromal enrichment of transforming growth factor beta 1 (TGFB1), supporting an immunosuppressive milieu (Fig. 5E). Gene set enrichment analysis highlighted activation of Notch, phosphoinositide 3-kinase–AKT serine/threonine kinase 1–mechanistic target of rapamycin and mitogen-activated protein kinase signalling pathways (Fig. 6). Spatial transcriptomics and RNA sequencing datasets are available in the NCBI Gene Expression Omnibus under accession No. GSE254830.

**Fig. 5.**
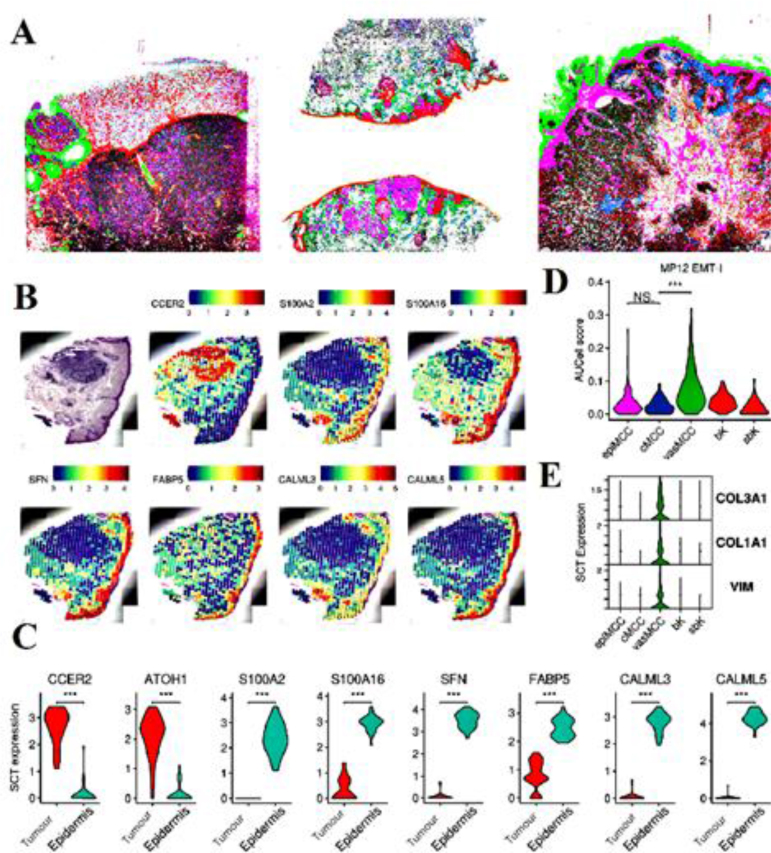
Spatial transcriptomic architecture of spontaneous Merkel cell carcinoma (MCC) in *Sciurus anomalus pallescens*. (A) Spatial transcriptomic profiling delineates discrete molecular clusters within the tumour microenvironment. Colour-coded clusters correspond to histologically distinct regions: magenta denotes tumour core enriched in neuroendocrine lineage genes (atonal basic helix–loop–helix transcription factor (ATOH1), insulinoma-associated protein 1 (INSM1) and sex-determining region Y-box transcription factor 2 (SOX2)); cyan indicates the invasive front with upregulated proliferative and immune checkpoint markers (marker of proliferation Ki-67 (MKI67), CD274 and cytotoxic T-lymphocyte-associated protein 4); yellow represents the peritumoural stromal niche expressing extracellular matrix and macrophage-associated genes (collagen type I alpha 1 chain (COL1A1), CD68 and C-X-C motif chemokine ligand 12 (CXCL12)). (B) HE reference image of the corresponding tissue section with matched spatial grid overlay. (C) Spatially resolved expression of neuroendocrine lineage markers (ATOH1, SOX2 and INSM1) defining the highly cellular tumour core. (D) Expression of proliferative and immune checkpoint genes (MKI67 and CD274) highlighting the invasive front and immune-modulated peripheral tumour zone. (E) Stromal compartment enriched for extracellular matrix and immune regulatory genes (COL1A1, CD68 and CXCL12), delineating the supportive peritumoural microenvironment. MP12 EMT-I – module-panel 12 epithelial–mesenchymal transition index; NS – not significant; VIM – vimentin; CCER2 – coiled-coil glutamate-rich protein 2; S100A2/16 – S100 calcium-binding protein A2/A16; SFN – stratifin; FAPB5 – fatty acid binding protein 5; CALML3/5 – calmodulin-like 3/5; SCT – secretin; COL3A1 – collagen type III alpha 1 chain; epiMCC – epithelial MCC cluster; cMCC – classic MCC cluster; vasMCC – vascular MCC cluster; bk – background tissue cluster; sbk – sub-background tissue cluster; Data are visualised as semi-transparent molecular overlays corresponding to tumour (magenta), invasive front (cyan) and stromal (yellow) clusters. Scale bar = 1 mm

**Fig. 6.**
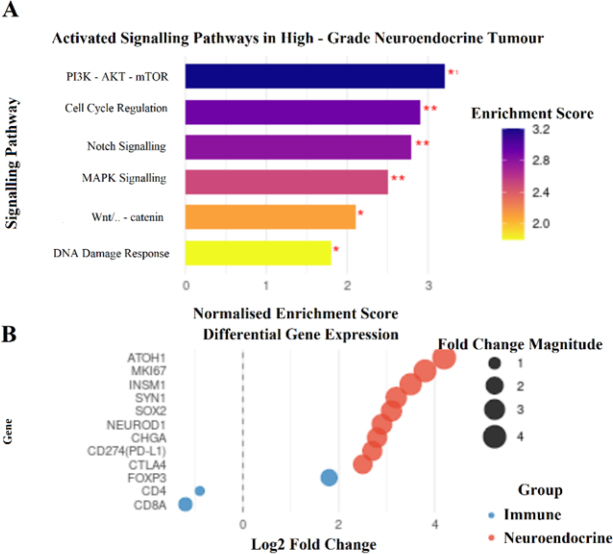
Analysis of *Sciurus anomalus pallescens* tissue for gene-set enrichment highlighting activation of Notch, phosphoinositide 3-kinase–AKT serine/threonine kinase 1–mechanistic target of rapamycin (PI3K-AKT-mTOR) and mitogen-activated protein kinase (MAPK) signalling pathways, consistent with a high-grade neuroendocrine tumour phenotype. Wnt/β-catenin – wingless+int–beta-catenin signalling pathway; ATOH1 – atonal basic helix–loop–helix transcription factor 1; MKI67 – marker of proliferation Ki-67; INSM1 – insulinoma-associated protein 1; SOX2 – sex-determining region Y-box transcription factor 2; NEUROD1 – neurogenic differentiation factor 1; CHGA – chromogranin A; PD-L1 – programmed-death protein 1; CTLA4 – cytotoxic T-lymphocyte-associated protein 4; FOXP3 – forkhead box subfamily P member 3

### Imaging mass cytometry and immune landscape

Imaging mass cytometry visualised distinct tumour-stroma boundaries, with proliferative tumour clusters surrounded by fibrocellular stroma (Fig. 6). Populations of CD8^+^ T cells were distributed predominantly at the tumour-stroma interface, forming dense peritumoural aggregates with minimal infiltration into tumour parenchyma (Fig. 7). Spatial neighbourhood analyses confirmed immune exclusion patterns, with increased mean nearest-neighbour distance between tumour cells and CD3-positive lymphocytes (Fig. 8). Quantitatively, the mean tumour-CD3^+^ T cell nearest-neighbour distance was 28.4 μm, compared with 8.2 μm within stromal regions (P-value < 0.01, Ripley’s K), supporting a statistically robust immune-tumour segregation phenotype. Spatial imaging datasets are archived in European Molecular Biology Laboratory European Bioinformatics Institute BioStudies under accession No. S-BSST1287.

**Fig. 7.**
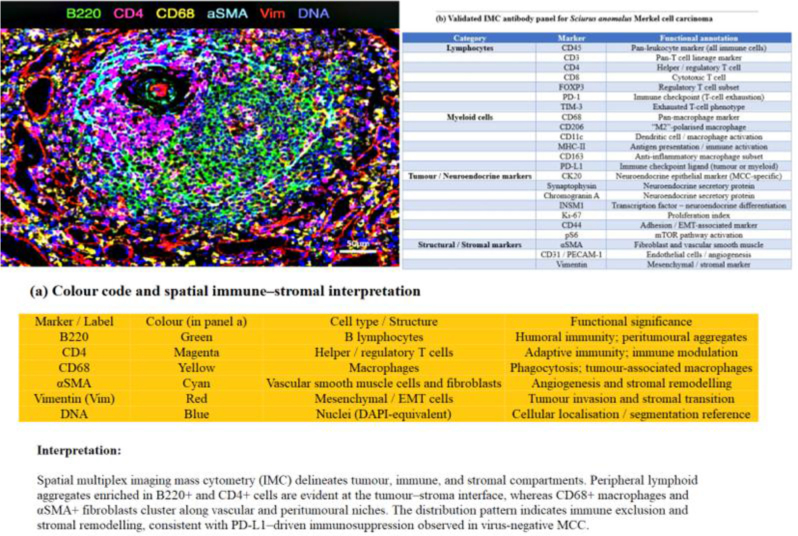
Integration of spatial transcriptomic data from *Sciurus anomalus pallescens* tissue with imaging mass cytometry revealing spatial juxtaposition – but not intratumoural colocalisation – of programmed-death ligand 1 (PD-L1)^+^ tumour cells and CD8^+^ lymphocytes, consistent with an immune-excluded phenotype. FOXP3 – forkhead box subfamily P member 3; PD-1 – programmed-death protein 1; TIM-3 – T-cell immunoglobulin and mucin-domain containing protein 3; MHC-II – major histocompatibility complex class II; MCC – Merkel cell carcinoma; INSM1 – insulinoma-associated protein 1; EMT – epithelial–mesenchymal transition; pS6 – phosphorylated ribosomal protein S6; αSMA – alpha smooth-muscle actin; PECAM-1 – platelet endothelial cell adhesion molecule-1; DAPI – 4′,6-diamidino-2-phenylindole

**Fig. 8.**
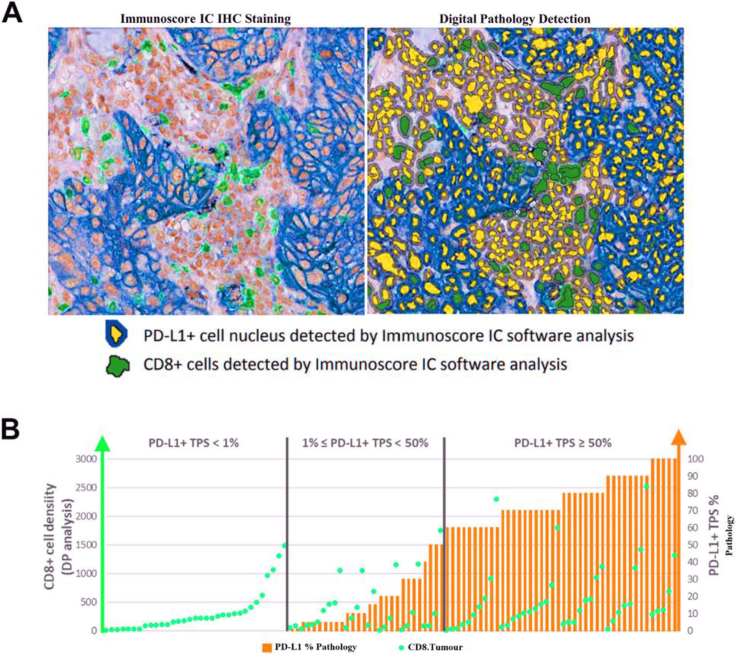
Spatial immune architecture and marker composition of spontaneous Merkel cell carcinoma (MCC) in *Sciurus anomalus pallescens*. (A) Spatial immune and stromal landscape within an MCC lesion showing B cells (B220, green), helper T cells (CD4, magenta), macrophages (CD68, yellow), vascular alpha smooth-muscle actin and fibroblasts (cyan), vimentin-positive mesenchymal cells (red) and nuclei (DNA stain, blue). A peritumoural lymphoid rim and tumour-associated macrophage clustering indicate immune exclusion and stromal remodelling consistent with programmed-death ligand 1 (PD-L1)-driven immune modulation. Scale bar = 50 μm. (B) CD8^+^ cell density and PD-L1 tumour proportion score (TPS) across cases stratified by PD-L1 expression level. IC – immune checkpoint; IHC – immunohistochemistry; DP – digital pathology

### Viral and molecular profiling

Unbiased metagenomic sequencing failed to detect Merkel cell polyomavirus (MCPyV) or other polyomavirus-related sequences. Viral metagenomic sequencing data are deposited in the NCBI Sequence Read Archive under BioProject PRJNA1015424 (SRA accession Nos SRR25843491–SRR25843492). A qPCR targeting the MCPyV T antigen yielded no amplification in tumour DNA, whereas positive controls amplified with Ct values of 18–22. Sequencing of RNA revealed marked upregulation of genes implicated in DNA damage response (ataxia-telangiectasia mutated (ATM) and checkpoint kinase 1 (CHEK1)), cell cycle regulation (CDK1, CCNB1) and neuroendocrine lineage differentiation (INSM1 and ATOH1). Mass spectrometry proteomics data have been deposited to the ProteomeXchange Consortium via the PRIDE partner repository with dataset identifier PXD05512.

**Fig. 9.**
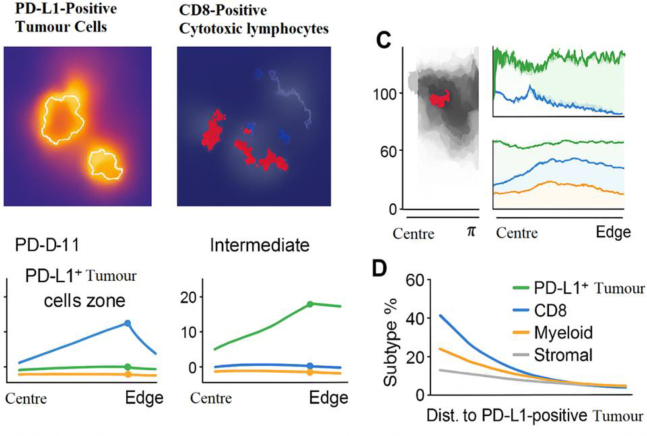
Single-cell segmentation and quantitative analysis of imaging mass cytometry data in *Sciurus anomalus pallescens* Merkel cell carcinoma tissue, showing a tumour to stromal cell ratio of 3.4 : 1, highlighting high cellularity and reduced immune infiltration. Panels C and D represent the spatial distribution of PD-L1-positive tumour cells and CD8-positive cytotoxic lymphocytes relative to the tumour centre and edge. PD-L1 – programmed death ligand 1

## Discussion

This report documents the first confirmed occurrence of MCC in the Caucasian squirrel (*Sciurus anomalus pallescens*) to the best of our knowledge. This animal is an IUCN Red List entry and a species for which spontaneous neoplastic conditions remain almost entirely undescribed (40). The tumour exhibited a constellation of histomorphological, immunophenotypic and molecular features that align closely with human and veterinary MCC, thereby establishing a robust cross-species diagnostic framework. The combination of classic small, round, blue cell cytomorphology, characteristic CK20 paranuclear dot-pattern staining and diffuse neuroendocrine marker expression provides compelling evidence for true neuroendocrine lineage differentiation, allowing differentiation from histologic mimics (11).

The absence of MCPyV represents a critical biological finding. Merkel cell carcinoma in humans arises through two major oncogenic routes: MCPyV-driven tumourigenesis and virus-negative carcinogenesis, the latter typically being associated with UV-driven mutational burdens and aggressive biological behaviour (18). In the present case, deep viral metagenomics, a targeted qPCR and spatial transcriptomic screening all failed to identify MCPyV sequences. The transcriptomic enrichment for DNA damage response pathways (ATM and CHEK1) supports a virus-negative oncogenic phenotype in the squirrel, suggesting that MCC in this species may follow a carcinogenic trajectory more similar to UV-associated or mutagen-driven MCC than to polyomavirus-mediated neoplasia (25).

Spatial transcriptomics and imaging mass cytometry provided significant insights into the tumour microenvironment. The sharply defined immune-excluded architecture, with T lymphocytes confined to perivascular and peripheral stromal domains, aligns with patterns observed in aggressive human MCC. Although major histocompatibility complex class I status was not directly assessed, the spatial segregation of T cells and the enrichment of TGFB1 are consistent with canonical immune-excluded MCC architecture (12). The suppression of CXCL9/10-CXCR3 signalling and stromal enrichment of TGFB1 identify key molecular mediators of lymphocyte exclusion. These findings are noteworthy because immune infiltration patterns in MCC carry major prognostic implications: tumours lacking intratumoural CD8^+^ T cells are associated with poorer clinical outcomes and reduced responsiveness to immunotherapy (1). Although little is known about immunobiology in *S. anomalus*, the parallels with human MCC suggest a comparable stromal-immune barrier mechanism.

From a conservation and wildlife pathology standpoint, the emergence of MCC in a near-threatened rodent species underscores the necessity of vigilant neoplastic surveillance in both wild and captive populations. Chronic environmental stressors, anthropogenic toxins or subclinical exposures could contribute to tumour initiation or progression in vulnerable wildlife hosts (26). This case also underscores the diagnostic challenges inherent to wildlife oncology. Antibody cross-reactivity and species-specific epitope variability necessitated a rigorous validation pipeline, aligning with recommended guidelines for cross-species immunohistochemical validation (33, 42).

In summary, this study provides a comprehensive multimodal characterisation of MCC in the Caucasian squirrel and establishes a diagnostic reference for future cases in small mammal species. The virus-negative phenotype, the immune-excluded microenvironment and the proliferative transcriptomic profile closely mirror high-risk human MCC biology. These findings contribute meaningful comparative oncology value and highlight the importance of integrating advanced spatial and molecular technologies into wildlife tumour diagnostics. Broader recognition of MCC across taxa may facilitate improved surveillance and refined understanding of neuroendocrine carcinogenesis in zoological species.

### Conservation implications

This case establishes a diagnostic framework for a highly aggressive cancer in an endangered rodent species, aiding wildlife veterinarians and pathologists in future cases. The virus-negative, immune-excluded phenotype suggests potential environmental or genetic drivers, highlighting the need for health surveillance in both captive and wild populations of susceptible species. Furthermore, the integrated use of spatial transcriptomics and imaging mass cytometry provides a powerful methodological blueprint for detailed tumour ecosystem analysis in wildlife, moving beyond traditional histopathology to inform conservation medicine.

## Conclusion

This integrated multimodal analysis confirms the first documented case of Merkel cell carcinoma in the endangered Caucasian squirrel (*Sciurus anomalus pallescens*). The tumour exhibited characteristic histomorphological and immunophenotypic features of MCC, including a high-grade neuroendocrine phenotype, a characteristic CK20 paranuclear dot pattern, and elevated proliferative activity. Molecular profiling revealed a virus-negative (MCPyV-negative) status, activation of DNA damage response and proliferative signalling pathways, and a distinct immune-excluded tumour microenvironment. These findings not only expand the comparative oncology spectrum of MCC but also demonstrate the utility of advanced spatial and molecular technologies – spatial transcriptomics and imaging mass cytometry – for comprehensive tumour ecosystem analysis in wildlife species. Future studies should investigate potential environmental or genetic drivers of oncogenesis in susceptible wildlife populations and explore the conservation implications of emerging neoplastic diseases in threatened species.

## Supplementary Material

Supplementary Material Details
