# Supplementary material for: Multimodal characterisation of spontaneous Merkel cell carcinoma in the endangered Caucasian squirrel (Sciurus anomalus pallescens): integrating spatial transcriptomics, imaging mass cytometry and metagenomic sequencing
Source: J Vet Res. 2026 Jun 30;70(2):321–34. doi: 10.2478/jvetres-2026-0034 (PMC13334306; doi:10.2478/jvetres-2026-0034)

Supplementary Materials

Western blot analysis CK20, synaptophysin and chromogranin A in tumour lysates- full-length uncropped blots

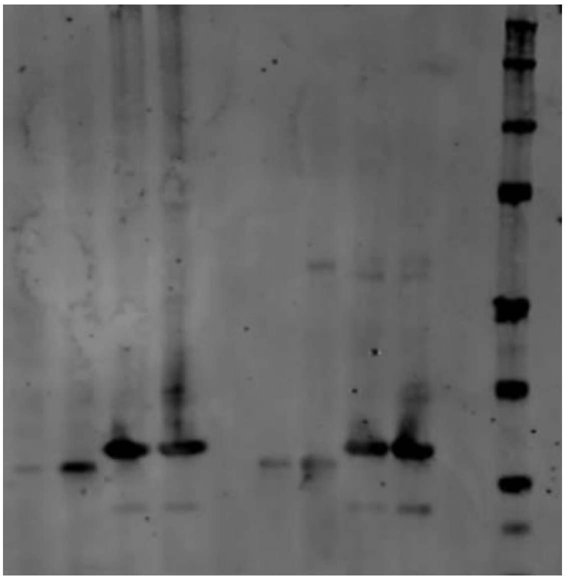

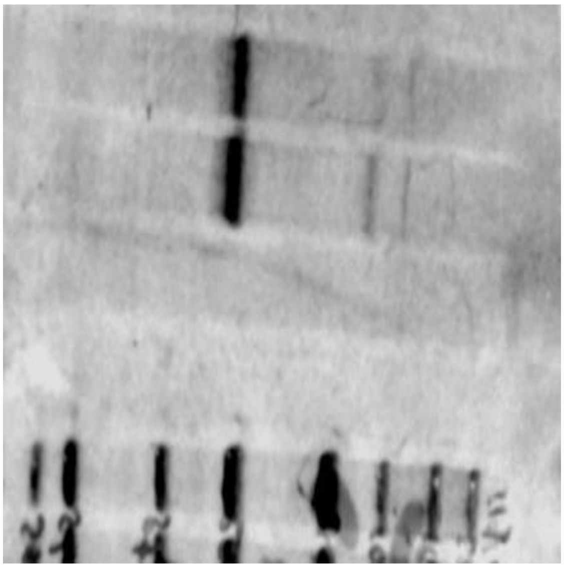

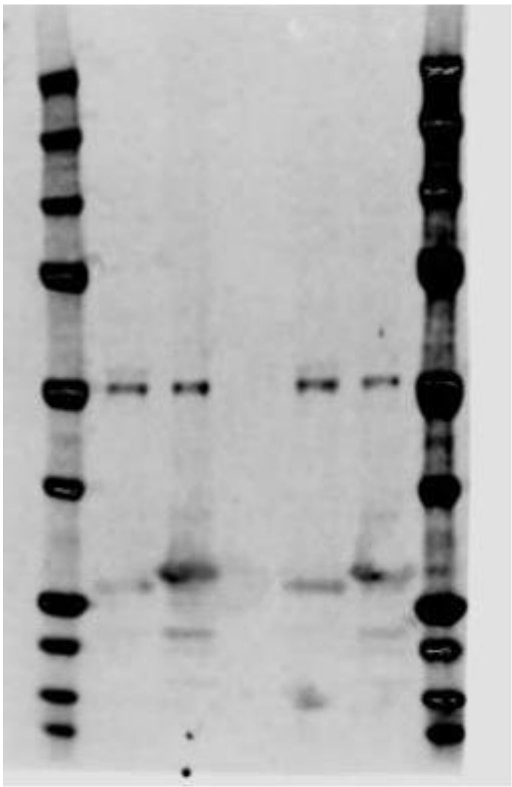

Supplement: Supplementary file 1 — Supplementary Material Details [file jvetres-2026-0034_sm.pdf]
